# Transcriptome profiling and validation of gene based single nucleotide polymorphisms (SNPs) in sorghum genotypes with contrasting responses to cold stress

**DOI:** 10.1186/s12864-015-2268-8

**Published:** 2015-12-09

**Authors:** Ratan Chopra, Gloria Burow, Chad Hayes, Yves Emendack, Zhanguo Xin, John Burke

**Affiliations:** Plant Stress & Germplasm Development Unit, Cropping Systems Research Laboratory, USDA-ARS, Lubbock, TX 79415 USA

**Keywords:** Cold stress, Sorghum, Transcriptome profiling, RNAseq, Single nucleotide polymorphism, Gene based variants

## Abstract

**Background:**

Sorghum is a versatile cereal crop, with excellent heat and drought tolerance. However, it is susceptible to early-season cold stress (12–15 °C) which limits stand-establishment and seedling growth. To gain further insights on the molecular mechanism of cold tolerance in sorghum we performed transcriptome profiling between known cold sensitive and tolerant sorghum lines using RNA sequencing technology under control and cold stress treatments.

**Results:**

Here we report on the identification of differentially expressed genes (DEGs) between contrasting sorghum genotypes, HongkeZi (cold tolerant) and BTx623 (cold sensitive) under cool and control temperatures using RNAseq approach to elucidate the molecular basis of sorghum response to cold stress. Furthermore, we validated bi-allelic variants in the form of single nucleotide polymorphism (SNPs) between the cold susceptible and tolerant lines of sorghum. An analysis of transcriptome profile showed that in response to cold, a total of 1910 DEGs were detected under cold and control temperatures in both genotypes. We identified a subset of genes under cold stress for downstream analysis, including transcription factors that exhibit differential abundance between the sensitive and tolerant genotypes. We identified transcription factors including Dehydration-responsive element-binding factors, C-repeat binding factors, and Ethylene responsive transcription factors as significantly upregulated during cold stress in cold tolerant HKZ. Additionally, specific genes such as plant cytochromes, glutathione s-transferases, and heat shock proteins were found differentially regulated under cold stress between cold tolerant and susceptible genotype of sorghum. A total of 41,603 SNP were identified between the cold sensitive and tolerant genotypes with minimum read of four. Approximately 89 % of the 114 SNP sites selected for evaluation were validated using endpoint genotyping technology.

**Conclusion:**

A new strategy which involved an integrated analysis of differential gene expression and identification of bi-allelic single nucleotide polymorphism (SNP) was conducted to determine and analyze differentially expressed genes and variation involved in cold stress response of sorghum. The results gathered provide an insight into the complex mechanisms associated with cold response in sorghum, which involve an array of transcription factors and genes which were previously related to abiotic stress response. This study also offers resource for gene based SNP that can be applied towards targeted genomic studies of cold tolerance in sorghum and other cereal crops.

**Electronic supplementary material:**

The online version of this article (doi:10.1186/s12864-015-2268-8) contains supplementary material, which is available to authorized users.

## Background

*Sorghum bicolor* (L.) Moench is a warm season species known for its drought tolerance and adaptation to high temperature but is susceptible to adverse effects of low temperature [1]. Sorghum originated in the semi-arid tropics and is generally sensitive to low-temperature stress and suffers cold injury when subjected to nonfreezing temperatures below 12–15 °C [2]. Low-temperature-induced inhibition of germination and emergence in the field followed by stunted growth is a manifestation of cold susceptibility problem in sorghum [3]. The inability to germinate prevents seedling emergence, resulting in poor stands, and subsequent growth of seedlings is hampered by cool air temperatures [1]. Field evaluations for cold tolerance require multi-environment or multi-year testing as presence of genotype x environment interactions complicates the interpretations [4]. Growth chamber assays for germination or vigor testing have been used to discriminate weak lines before planting and have been useful in quantifying cold tolerance in sorghum lines [5], but needs to be verified under field conditions as well [1].

Genetic variability for cold tolerance have been reported and identified within sorghum germplasm pools. Sources of cold tolerance are landraces that have evolved in the temperate regions of China [6] and have exhibited higher seedling emergence and improved seedling vigor under cool conditions compared to select US hybrids and elite inbreds [7]. Dissecting the cold tolerance traits in these landraces would be highly beneficial to efforts of transferring tolerance traits into the elite germplasms by backcrossing. To identify the genome loci associated with cold tolerance, QTL studies for cold germinability and seedling vigor have been carried out [1, 8, 9]. Molecular markers such as SSRs have been utilized for identification of QTLs for cold tolerance. However, there is still scant information that will provide an understanding of cold response and tolerance in sorghum as compared to other warm season crops like rice.

As early-season cold tolerance is a complex trait, it is important to explore the overall dynamics of gene expression and transcriptome during cold stress to facilitate an understanding of response to cold stress. The effects of low temperature stress at the molecular level through genome-wide gene expression analysis were reported in other warm season cereal crops such as rice [10], but have not been applied to sorghum. The use of advance genomic sequencing approach to examine tolerance mechanisms to cold stress in sorghum could facilitate better understanding of the molecular genetic basis and be an effective complement to enhance overall sorghum stress tolerance.

Recently, the use of next generation sequencing approaches such as RNAseq technology was proven highly valuable in expanding molecular genetic studies as it could be applied simultaneously to generate the bi-allelic variant information. The large amounts of sequence information from next generation sequencing technologies can be annotated to examine the role of specific transcripts under abiotic stress. Transcriptome profiling using sequencing technologies have been performed in sorghum under nitrogen stress, and osmotic stress [11, 12] and combined heat and drought stresses. These studies provided information on specific genes involved in defense mechanism against specific abiotic challenges. However, there is scarce information on transcriptome profiling for sorghum cold stress analysis and the identification of SNPs and variants that could regulate complex traits such as cold tolerance. The SNPs obtained from these studies could be further utilized in performing the QTL studies, facilitating to target the genic marker controlling the trait.

There is critical need to learn more about cold tolerance mechanism as new areas for sorghum cultivation in cooler northern latitudes are expanding. Generating gene-specific SNPs between the cold tolerant and susceptible sorghum genotypes would help in targeting loci which influences the traits directly and will aid in expediting breeding for cold tolerance. The objectives of this study were (a) to determine variation/changes in gene expression between cold tolerant and sensitive sorghums, (b) to identify and validate genic SNPs between cold tolerant and sensitive and (c) to generate a new genetic resource of genic SNPs that can be used as markers for cold stress QTL studies.

## Results

### Variation for cold tolerance response of sorghum genotypes and transcriptome profiling approach

**Fig. 1:**
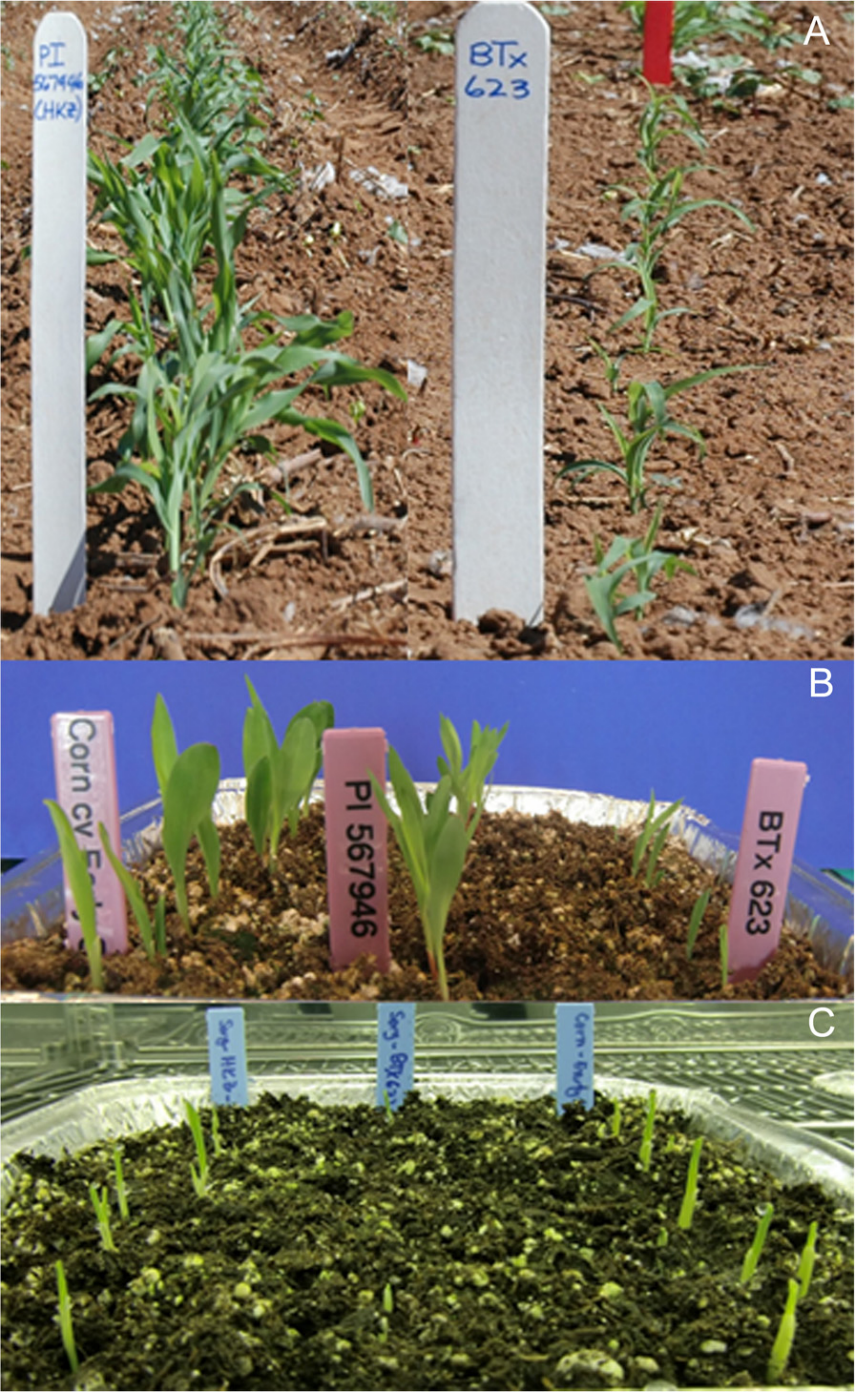
Description of BTx623 and HKZ germplasm and their differential response to cold stress

Sorghum germplasm BTx623 and HKZ were previously selected based on the 72 h cold germ test, field emergence and seedling vigor (Fig. 1). HKZ genotype exhibited higher germination and greater than 60 % field stand under field conditions during cool early season sowing compared to BTx623. In terms of seedling growth, HKZ displayed vigorous seedling growth as compared to BTx623 at 7 days after sowing at 14 °C.

Transcriptome profiling from whole seedlings was performed by quantifying the gene expression between the two germplasm under control conditions and cold stress separately (Additional file 1). cDNA libraries made from the total RNA were sequenced using massively parallel technologies, and short reads (50–80 nucleotides) obtained were further processed for expression analysis and SNP calling.

### Transcriptome analysis in response to cold stress in sorghum

**Fig. 2:**
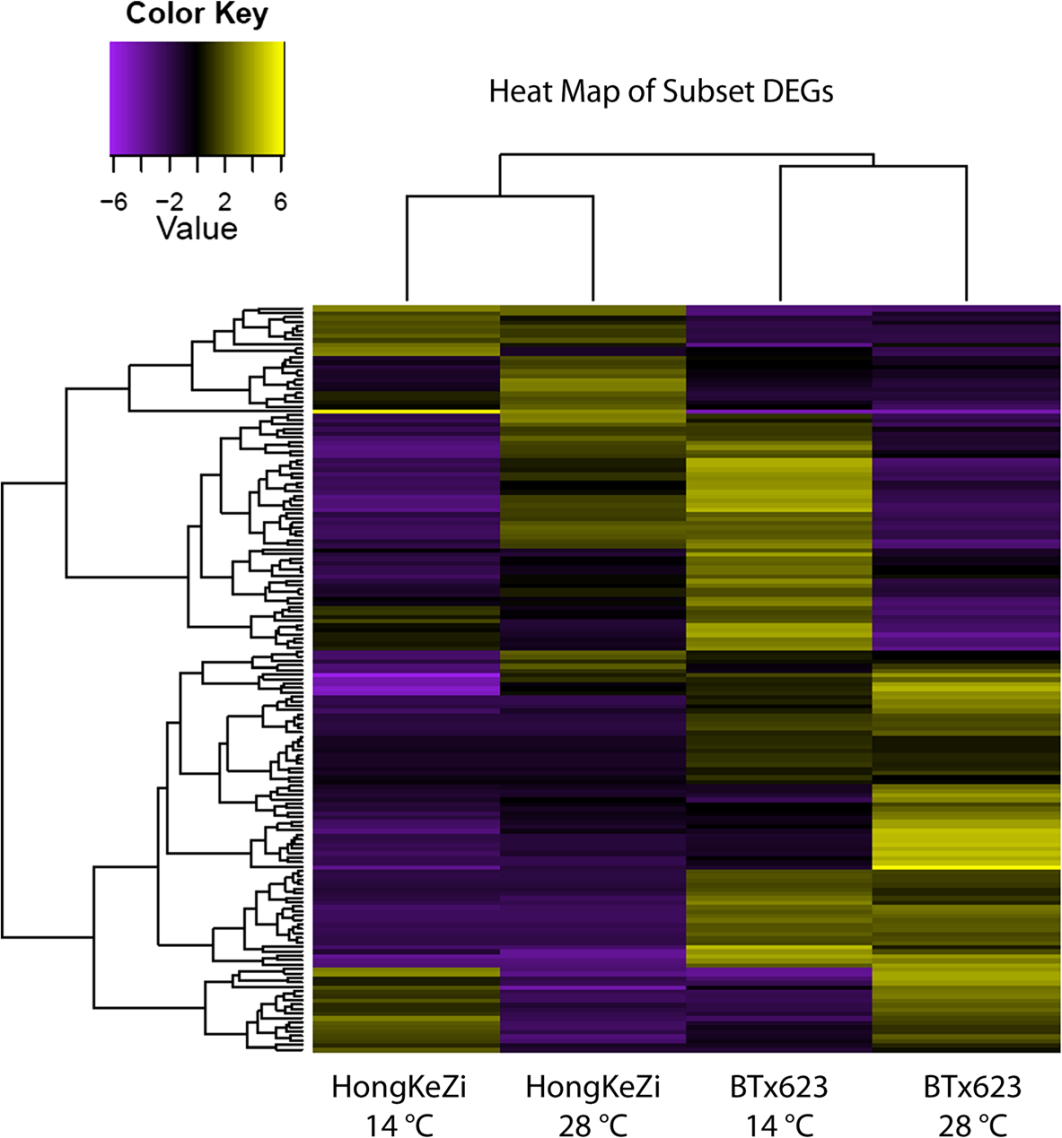
Heat map of differentially expressed genes among the sorghum genotypes under normal and stress conditions

The raw sequences were aligned to the sorghum reference genome v. 1.0 using GMAP resulting in approximately 77.3 % coverage of the sorghum transcriptome. To compare differential expression patterns among four libraries (Table 1), we normalized the tag distribution for gene expression level in each library to come up with an effective library size and subsequently extracted the differentially expressed transcripts using the criterion *p* value ≤0.05 and log2 fold-change ≥2 using edgeR [13] according to the user manual. This provided an empirical approach and eliminated the bias introduced by RNA composition. For the four libraries developed, our comparative analysis showed that a range of 625–1033 transcripts displayed significant change in expression based on abundance. Specifically, the differential expression patterns among libraries revealed that about 625 transcripts showed significant changes for BTx623 between 14 and 28 °C. For HongKeZi, approximately 344 transcripts showed differential expression between 14 and 28 °C. When the two genotypes were compared at 14 °C, 751 DEGs were identified and approximately 407 DEGs were detected between the genotypes at 28 °C (Table 1, Fig. 2).

**Table 1:** Numerical counts of genes with two-fold or > expression between the sorghum genotypes under normal and stress conditions at *P*-value of 0.001, Normal: 28 °C after 7 days and Stress: 14 °C after 7 days

	BTx623_14C_7	BTx623_28C_7	HKZ_14C_7	HKZ_28C_7
BTx623_14C_7	0	625	751	833
BTx623_28C_7	625	0	1033	407
HKZ_14C_7	751	1033	0	344
HKZ_28C_7	833	407	344	0

### Annotation and gene ontology of Sorghum DEGs during cold stress

**Fig. 3:**
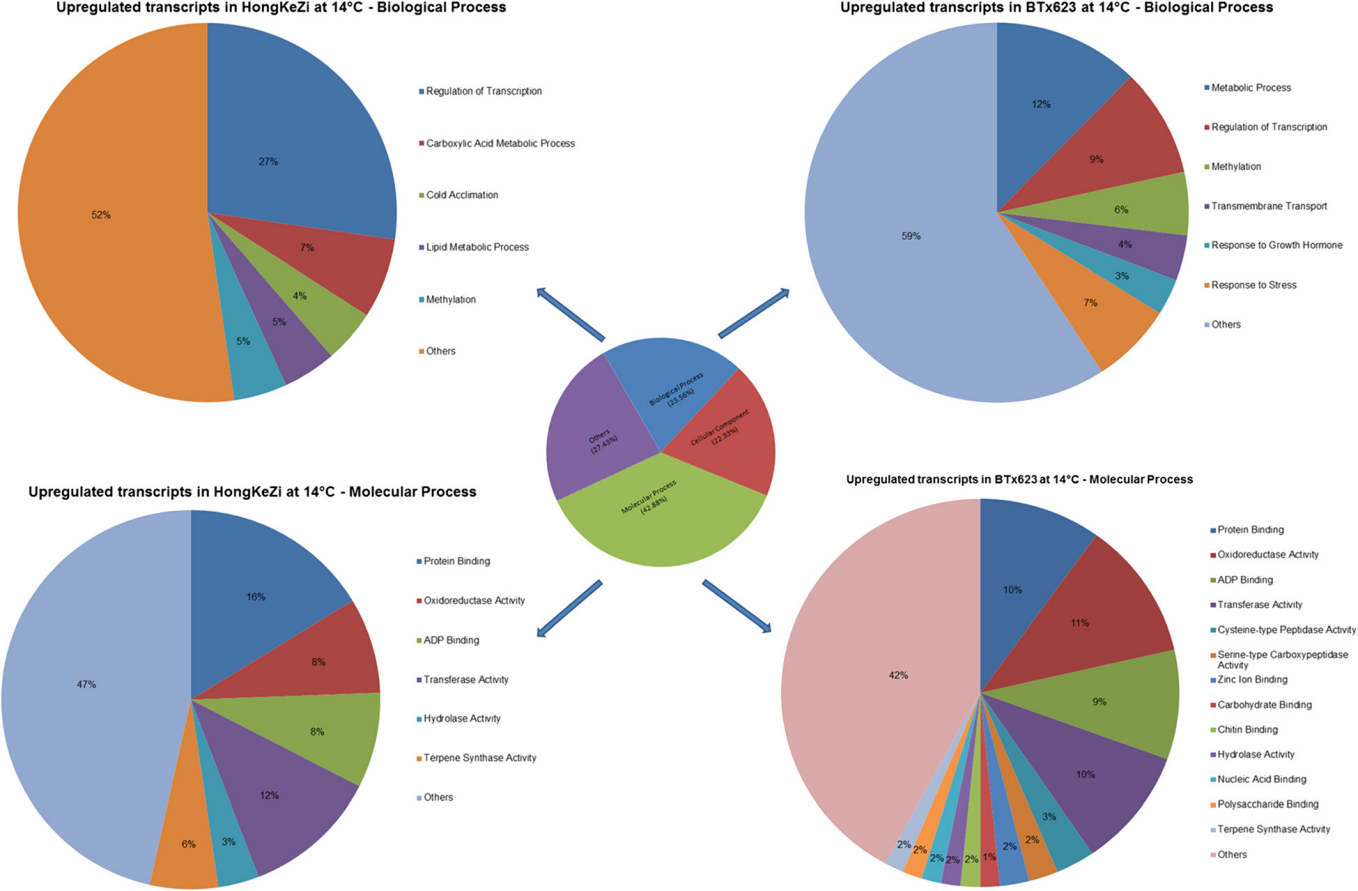
Differentially expressed genes assigned to GO domains

The differentially expressed genes were further compared with the annotated genes of the sorghum genome (http://phytozome.jgi.doe.gov/). The gene description, GO annotations were retrieved for each of the DEGs and were used for downstream interpretation of datasets. Differentially expressed genes were assigned to different GO domains and about 27.4 % expressed transcripts were not assigned to any of the categories (Fig. 3).

To streamline the analysis, the upregulated transcripts in HongKeZi and BTx623 at 14 °C were categorized to molecular and biological processes (Fig. 3). Approximately 8–11 % of the upregulated transcripts under cold stress were involved in the oxido-reducatse activity for HKZ. About 4 % of the DEGs were annotated as transcripts with putative role in cold acclimation for HongKeZi grown at 14 °C. Meanwhile, only about 3 % of the transcripts in cold susceptible line exhibited changes in category for stress responsive genes.

### Comparative analysis of a subset of DEGs under normal and cold stress conditions

**Fig. 4:**
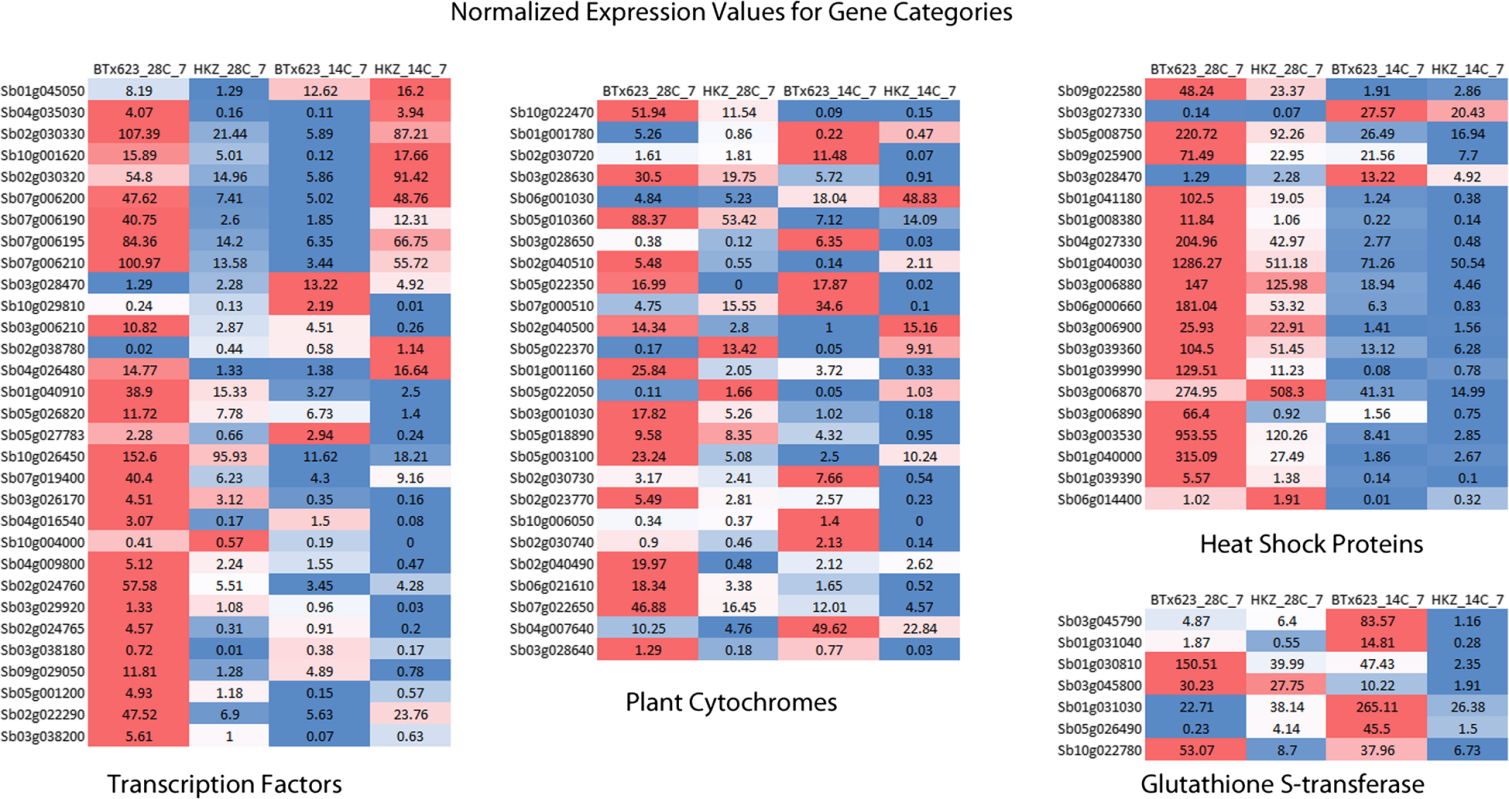
Normalized FKPM values for the genes that were differentially regulated among genotypes for some genes of interest

We explored the RNAseq results for previously reported genes related to temperature stress responses in sorghum and related cereal crops and use these as bases for establishing a subset of DEGs for further analysis (Fig. 4). For example, the AP2 family transcription factors such as *Sb10g001620* (CBF5) and *Sb02g030330* (CBF6) showed relatively high abundance in HKZ under cold stress treatment. Notably, a transcription factor previously reported to be associated with cold-stress, Ethylene responsive transcription factor (ERF) also exhibited high abundance in the tolerant genotype under cold stress. The general trend observed in this study indicated that transcripts which showed higher abundance under stress in tolerant line had relatively low abundance of transcripts under control condition.

However, we also found that a number of genes including the heat shock proteins/transcription factors showed low abundance in both susceptible and tolerant genotypes under cold stress compared to control condition. Also, a number of genes involved in detoxification of xenobiotics like Glutathione-S-transferase were observed to be abundant in susceptible genotype mainly under cold stress. Specifically, in the sensitive genotype, *Sb01g031040* similar to (Glutathione S-transferase GST 31) and *Sb01g031030* showed similar relative abundance under control condition but were upregulated under cold stress (Fig. 4). Likewise, genes for plant cytochromes were also differentially expressed between the genotypes studied here. *Sb06g001030* (P450 CYP99A1), *Sb02g040500* (P450 CYP709C1) were observed to be upregulated in HKZ under stress, while *Sb02g030730* (P450 like also) and *Sb05g018890* exhibited higher relative abundance in BTx623 under cold stress (Fig. 4).

### Quantitative RT-PCR of select DEGs under cold stress

To confirm the gene expression profiling data obtained from RNA-seq, we performed qRT-PCR analysis to evaluate the expression of five selected candidate genes (Additional file 2). The gene specific primers used are listed in Additional file 3. For the five genes evaluated, the differential expression or qRT-PCR results agreed with RNAseq analysis which indicated that RNAseq results can be confirmed with qRT-PCR data. This aspect was explored primarily for technical validation of the RNAseq analysis.

### Identification and validation of gene based variants

**Fig. 5:**
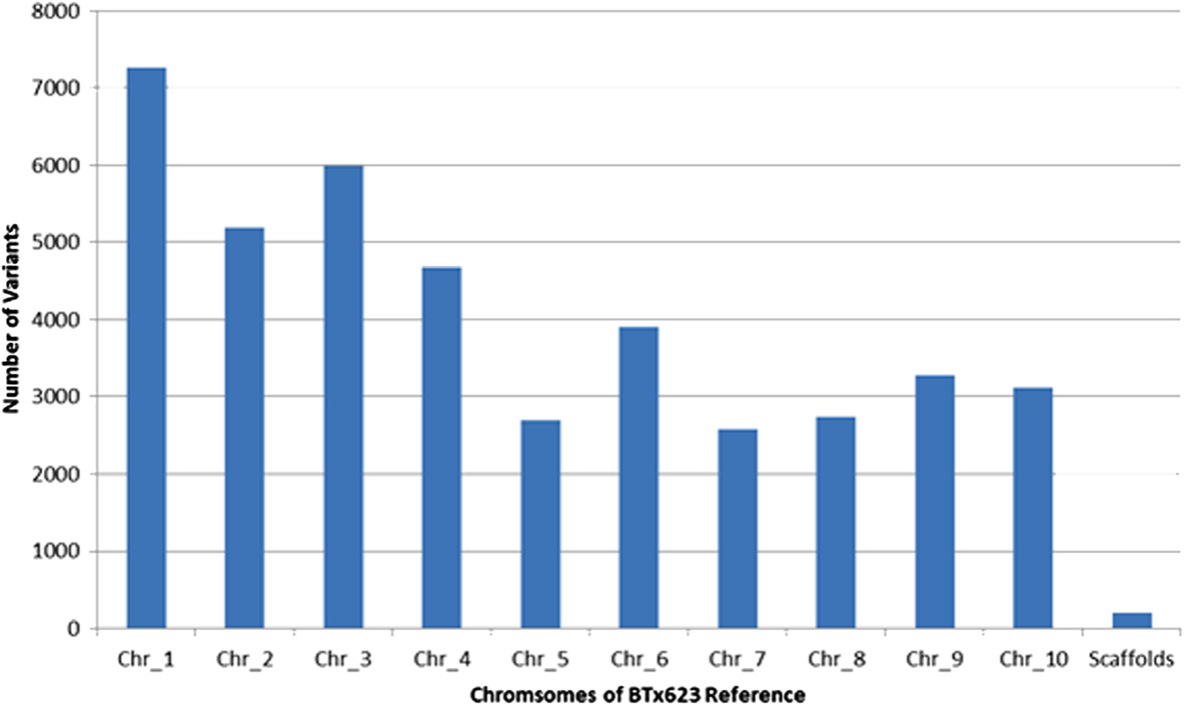
Distribution of number of variants on each of the Sorghum chromsomes

We conducted further analysis of the RNAseq data through identification of biallelic variants between the two genotypes studied. The corresponding SNPs and insertion deletions (indels) were identified by comparing the resulting variant calls to the custom reference genome sequence using the Alpheus software [14] with the parameters: variant allele frequency ≥10 %, variant unique reads ≥2 and average base quality score ≥20 in the given accession, and either no variant allele or any allele present in the other accession(s). The stringency was increased thereafter to limit the number of unique reads to four. From this analysis, the total number of SNP plus indel variants identified was 41,603 (Additional file 4). To obtain an estimate of the genomic distribution of variation, we calculated that the average number of total variants in each chromosome was approximately 4140 (Fig. 5). The most number of variants were found in chromosome 1, while the least was found in chromosome 5 and 7. These results may be reflective of the number of genes annotated in each chromosome.

We assessed a set of 114 SNPs using allele-specific assay chemistry/endpoint genotyping approach to validate the bioinformatics SNP call (Additional files 5 and 6) using BTx623, HKZ and two other sorghum genotypes RTX430 and GaigaoLiang (PI 610727 (Additional file 7). The two other sorghum genotypes, RTx430 and GGL were included at this point to assess if the variation can be verified in other sorghum germplasm.

Initially, we evaluated three SNPs from each chromosome and specific primers compatible with KASP assay using Primer3 (Additional file 6). The selected 30 gene based SNPs and results of genotyping including the amino acid changes resulting from the SNP variation between genotypes are described in Additional file 8. The rest of the 84 SNPs assayed for validation are given Additional file 5. Ninety percent of the assays validated the bioinformatic allele calls between BTx623 and HKZ (Additional file 8). Genotype call for RTx430 and GGL indicated greater SNP polymorphism between GGL and BTx623 (reference genome) than between RTX430 and BTx623 (Additional files 5 and 8).

### Development of an integrated database combining transcriptome profile and gene based SNPs

**Fig. 6:**
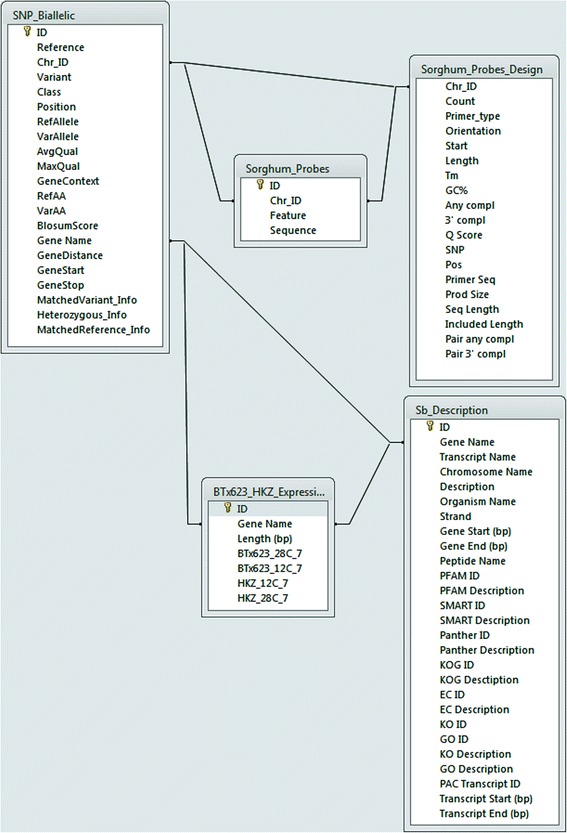
Overview of the database for retrieving the information on cold tolerant and sensitive lines

In this study we also organized the RNA-Seq data on expression profile and gene based SNP information from the cold sensitive and cold tolerant sorghum genotypes to generate a searchable database (Fig. 6, Additional file 4). The beta version of the database consists of the SNP information between two lines along with their features, expression of the cold stress (Additional file 1) and annotations retrieved from Phytozome for the transcripts (Additional file 9). Additionally we also included information on successful primers designed for the SNPs between the two contrasting genotypes (Additional files 10 and 11). We integrated these different files using Microsoft Access to develop the proposed integrated database as a resource for sorghum cold stress genomics research. The database is available from Cropping Systems Research Laboratory, USDA-ARS, Lubbock, TX, website (http://www.csrl.ars.usda.gov/psgd/index-sorghum.aspx.html).

## Discussion

Advances in novel high-throughput sequencing technologies provide immense opportunities to explore the molecular basis of response to abiotic stress, especially resistance and signaling-associated genes in different species by either *de novo* assembly or direct mapping to reference genome, resulting in identification and analysis of global transcriptome [13, 15]. Previous expression studies on biotic and abiotic stress in sorghum using microarray and next-generation technologies have shown greater promise of understanding genome-wide expression patterns of genes in sorghum [11, 12, 16]. Expression profiling of plant responses using next-generation sequencing (RNA seq) can be effective way for understanding genes and pathways associated with cold stress response and expression of tolerance for this specific stress in sorghum.

The genome size of sorghum is estimated to be 726,616,606 base pairs, arranged into 10 chromosome pairs or 2n = 20 based on latest bioinformatics v2.1 analysis (http://phytozome.jgi.doe.gov/). Approximately 39,441 protein coding transcripts were annotated from the whole genome sequence of sorghum (http://phytozome.jgi.doe.gov/). The current study provides ~77 % coverage of the sorghum transcriptome. This coverage is comparable to that reported by Dugas [11] and Gelli [12]. It is assumed that the rest of the transcriptome was not covered due to rare transcript reads. Further, it was shown before that the success of covering the entire transcriptome is affected by tools or software’s used for alignment, and on the technology or chemistry used for sequencing [17].

Majority of the differentially expressed transcripts under biological process category identified in cold tolerant line HKZ were involved in the oxido-reductase activity, however only 3 % of this class of transcripts showed changes in the susceptible line, BTx623, (Fig. 3). Previous work have shown that oxidoreductases are involved in cold adaptation in rice [10]. These results indicates that cold acclimation transcripts found in other species such as rice are also differentially regulated in cold tolerant genotype HKZ and could be important in the expression of overall cold tolerance displayed by this genotype.

In this study, we observed that expression of transcription factors (TFs) under cold stress followed a pattern of up-regulation in the cold stress plants of HKZ as compared to BTx623 (Fig. 4). As an example, the relatively high abundance of transcription factors such as C-repeat binding factors (CBFs), ethylene responsive transcription factors (ERFs) from the APETALA2/Ethylene Responsive Factor (AP2/ERF) transcription factor family were upregulated in HKZ. Noteworthy is that the transcription factors belonging to the AP2/ERF family are conservatively widespread in the plant kingdom [18]. These transcription factors have been extensively studied in *Arabidopsis* and rice and were shown to be over-expressed under abiotic stress conditions including cold stress challenge [19]. Members of the DREBs/CBFs subfamily are rapidly induced in response to cold stress and, when over-expressed, improve tolerance to freezing [20, 21]. Transcription factors represent ideal targets for traditional or genetic engineering-assisted breeding of plants with specific traits related to stress tolerance. In particular, ERF transcripts are among the most interesting TFs because they have been selected through evolution to regulate a series of stress-response pathways.

We also observed that *Sb06g001030* (P450 CYP99A1), *Sb02g040500* (P450 CYP709C1), annotated as plant cytochrome like transcripts were relatively abundant in the tolerant lines under cold stress indicating possible involvement in defense mechanism (Fig. 4). Plant cytochromes P450s catalyze oxidation of a wide range of chemical reactions by activating dioxygen [22] and were reported to play an important role in response to stress [12, 23]. P450s (CYPs) are ubiquitously distributed and are involved in the biosynthesis of plant hormones and secondary metabolites, and CYPs accumulate in response to cold stress [24, 25].

We also observed that *Sb03g027330* gene was highly abundant in both genotypes under stress and *Sb03g028470,* another heat shock transcription factor transcript was relatively abundant in the susceptible genotype only under stress (Fig. 4). This relative abundance of HSFs and HSPs under cold stress could suggest a mid-point involvement in cold stress response between multiple stress pathways similar to observations made by Swindell et al. [26] and indicate that HSFs could have other functional features apart from heat shock responses. A number of studies indicated that heat shock proteins (HSPs) and transcription factors (HSFs) are involved in cellular response to various forms of stress besides heat. In *Arabidopsis* and other plant species, various Hsps have been induced by low temperature [27], osmotic stress [28], and salinity stress [29].

Notably, the transcript encoding Glutathione-S-transferase (GST) were also abundant in BTx623 under cold stress (Fig. 4). Glutathione S-transferases (GSTs) have functions in detoxification of various xenobiotic compounds and oxygen radicals [30]. Their roles in these biochemical pathways make them useful markers in the detection of stress in plant metabolism. An increased production of reactive oxygen species is a common consequence in plants under abiotic stresses, which usually damage cellular membranes and other cellular components resulting in oxidative stress and eventually cell death [31, 32]. It is likely that a high abundance of GSTs may protect sorghum cells from oxidative stress that is prominent in cold sensitive genotypes.

The whole genome identification of SNPs for a number of sorghum genotypes was performed previously using whole genome re-sequencing [33] or RADSeq technology [34]. Here we report that sequencing of transcriptome was useful in identification of a large number of genic SNPs. Genic SNPs and variations in structural genes are important as they have been documented as underlying genetic basis for many important agronomic traits. As an example,variation underlying an actual QTL controlling cold tolerance in the form low temperature germinability in rice was identified to be due to variation in a transcript that cause tissue weakening that allows protrusion of coleoptile during germination [35]. The number of SNPs that distinguished the two contrasting sorghum genotypes studied here suggests the strong potential of genic-SNPs for use as genetic markers for QTL or genome wide association studies. We suggest that the use of genic SNPs could be advantageous over conventional markers SSRs, RFLPs and RAPDs for targeting the loci controlling the complex traits such as cold, drought or salt stress by directly pointing to specific genes. From sequencing cDNA libraries of two contrasting parents we could identify ~41,000 SNPs that can aid in generating a new genetic resource for the sorghum community. The SNPs selected for validation indicated that the approach of sequencing cDNA would be beneficial for generating new set of DNA markers in sorghum. The SNP markers developed between the two contrasting genotypes for cold tolerance could be beneficial for identifying QTLs in early-season cold tolerance of sorghum.

The complex inheritance of abiotic-stress traits limits the genetic modification of plants based on a single gene to achieve satisfactory level of tolerance. Therefore an understanding of the molecular mechanisms of plant responses to different stresses is critical for manipulation of associated pathways that can enhance abiotic stress tolerance. Here we showed that the information from transcriptome profiling can useful as basis for characterization of germplasm exhibiting cold tolerance traits.

## Conclusion

Identification of common DEG transcripts between sorghum genotypes with contrasting stress tolerance would facilitate a better understanding of the genetic bases of cold tolerance. Here, Illumina RNAseq analysis demonstrated that gene transcripts involved in abiotic stress response, and defense mechanism were regulated in both tolerant and sensitive lines. Transcripts with higher expression could be useful as a biomarker for selection of tolerant/sensitive genotypes under cold stress conditions. The DEGs from tolerant genotypes would be the potential candidates to study further for improving cold tolerance of sorghum and related cereals. Finally, this study took advantage of the bi-allelic variation observed between the genotypes which were translated and validated into easy to use and accessible gene-based SNP markers for downstream genetic analysis of cold stress tolerance in sorghum.

## Methods

### Plant materials and cold treatment

Two sorghum germplasm, (BTx623 and HongKeZi), with contrasting response to cold stress under controlled and field conditions were used in the study. HongKeZi (PI567946, www.ars-grin.gov) is a cold tolerant line classified under the Kaoliang-nervosum working group identified by Franks et al., [7]. BTx623, a combine type inbred line classified under the kafir_zera-zera working group, is sensitive to cold stress under field conditions and served as the cultivar used in reference genome sequence (http://phytozome.jgi.doe.gov/) for sorghum.

For this study 20 seeds for each of the cultivar were planted in ragdoll set up under control and cold stress temperatures. The temperatures were continuous 14 °C for cold stress and 28 °C for control temperature using Conviron walk-in growth cabinets. Each temperature was represented by three replicates. The seedlings were allowed to grow under 14 h light/10 h dark conditions. The plants were grown in each temperature for 7 days. Each replicate was represented by five uniform seedlings (shoots and roots).

### RNA extraction and sequencing

Tissue samples for RNA extraction were obtained from five seedlings pooled together per replicate. Two replicates were extracted separately using the TRIzol reagent and company recommended protocols (Life Technologies, Grand Island, NY). The total RNA samples from each replicate were quantified using Nano Drop and subsequently purified using the RNAeasy mini clean up kit (Qiagen, Valencia, CA). The quality and quantity of RNA were examined using an Agilent 2100 Bioanalyzer (Agilent Technologies, Santa Clara, CA). Subsequent cDNA library construction and bar-coding for each of the samples from the two genotypes under each temperature treatment was conducted at the National Center for Genome Resources. RNA sequencing was performed on a GAIIx Analyzer (Illumina, San Diego, CA).

### Expression analysis and validation by qRT-PCR

The short reads were mapped against the Sorghum bicolor 79 genome (http://phytozome.jgi.doe.gov/) using GSNAP [36], allowing up to two mismatches. The number of reads in genes was counted and then, the edgeR package [37] with TMM normalization method was used to align expression values to a common scale. The fragments per kilo base per million (FPKM) values were also calculated for all the genes and considered as the expression level. The resulting expression values were log2-transformed. Average log signal values for each sample were then computed and used for downstream analysis. The cutoff of log2-fold value ≥2 (4-fold absolute value) and adjusted *P*-value <0.001 (FDR) were used for determining significant DEG transcripts. Pair-wise comparisons among the four libraries were conducted by comparing the sequenced samples to find common DEG transcripts across all genotypes. In addition, tolerant and sensitive genotypes were also compared among themselves and then to each other to asses if the differences in gene expression between sensitive and tolerant genotypes found are significant among the sorghum genotypes.

To evaluate the expression differences observed, qPCR primers were designed for five select targets using primer3 (Additional file 1). Briefly qRT-PCR was performed using copy-DNA libraries generated from the total RNA used for sequencing using Invitrogen cDNA synthesis kit (Invitrogen, Grand Island, NY). QPCR was performed on the cDNA of the four sequenced samples using SybrGreen on LightCycler 480.

### Gene annotations and GO analysis

The transcripts that are differentially expressed among samples with different temperature treatments were queried against the genome reference (www. phytozome v. 9.0) using Biomart interface at www.gramene.org. The specific gene features such as gene descriptions, GO domains, KEGG descriptions for each of the differentially expressed genes were retrieved from the database. This analysis allowed us to determine the major biological functions of DEGs.

### SNP identification and primer design

An Alpheus [14] sequence variant detection pipeline was used for read mapping and calling SNPs. SNPs and in-dels were identified with the variant allele frequency ≥10 %, variant unique reads ≥2 and average base quality score ≥20. The bi-allelic SNPs and in-dels identified were further filtered to minimum unique variant read of ≥4. Only bi-allelic SNPs were further used for the validation experiments. About 150 bp upstream and downstream of the SNP loci were extracted from the available genome sequence for use in primer design. These sequences were passed through primer3 program with the option of allele-specific and flanking primer method and the size range of 60–100 nucleotides (http://probes.pw.usda.gov/cgi-bin/batchprimer3/batchprimer3.cgi).

### Validation of SNPs using allele-specific assays

To validate biallelic SNPs Additional file 8 and test their occurrence in various sorghum genotypes, genomic DNA from four genotypes BTx623, HKZ along with RTx430 and PI 610727 (GGL) was isolated from leaf samples using modified CTAB method. End point genotyping using Kompetitive Allele Specific (KASP, LGC Genomics) chemistry was utilized according to manufacturer’s protocols in a LightCycler 480 (Roche, Branford, CT). Briefly, the assay mix consis of a final volume of 10 μl containing 1× KASP Reaction Mix (LGC Genomics, Hoddesdon, UK), 0.14 μl Assay mix, and 10–20 ng genomic DNA. The following cycling conditions were used: 15 min at 94 °C; 10 touchdown cycles of 20 s at 94 °C, 60 s at 63–55 °C (dropping 0.8 °C per cycle); and 30 cycles of 20 s at 94 °C, 60 s at 55 °C. Fluorescence detection of the reactions was performed using a built-in scanner and the data were analyzed using the LightCycler 480 software (Roche, Branford, CT).

### Development of an integrated database

The information obtained from above RNA seq and bioninformatic analysis for DEGs were compiled for transcript features such as gene and KEGG descriptions from Biomart (http://ensembl.gramene.org/biomart/martview/). The corresponding nucleotide polymorphisms observed between two genotypes were also compiled an individual datasheets. The primers designed for select SNP targets were recorded in a separate file. Subsequently the sorted files of information generated from transcriptome sequencing and gene based SNP identification were interlinked into a single database using Microsoft Access available for public use at (http://www.csrl.ars.usda.gov/psgd/index-sorghum.aspx.html).

## Additional files

Additional file 1:
**Differentially expressed genes/transcripts in at least one sample with 2 fold change and at p-value of 0.001.** (XLSX 162 kb) Additional file 2:
**Quantitative PCR plots of the selected expressed genes for technical validation.** (TIF 114 kb) Additional file 3:
**Primer sequences used technical validation of expression data obtained from RNASeq.** (XLSX 9 kb) Additional file 4:
**Variant information between BTx623 and HongKeZi and features of each mutation on the physical position.** (CSV 8445 kb) Additional file 5:
**Primer sequences and validation of additional variants using KASP technology.** (XLSX 14 kb) Additional file 6:
**Primer sequences for validation of bi-allelic variant calls.** (DOCX 15 kb) Additional file 7:
**An example of validation of SNPs between the sequenced accessions using KASP technology.** (TIF 307 kb) Additional file 8:
**Description of the SNP position and SNP features on the sorghum chromosomes and validation of the bioinformatics allele calls on the sequenced lines and in two additional sorghum Germplasm RTx430 and GGL (PI567946).** (DOCX 17 kb) Additional file 9:
**Description of transcripts retrieved from** ***sorghum bicolor*** **genome v 1.0.** (XLSX 7247 kb) Additional file 10:
**Primer sequences for selected variants to be used in building of integrated database.** (XLSX 5540 kb) Additional file 11:
**Sequences near SNP site to be used for primer design.** (XLSX 4098 kb)

## Abbreviations

bp: Base pair
CTAB: Cetyl trimethyl ammonium bromide
DEGs: Differentially expressed genes
GO: Gene ontology
KASP: Kompetitive(©LGC Genomics) allele specific PCR
KEGG: Kyoto encyclopedia of genes and genomes
nt: Nucleotide
PCR: Polymerase chain reaction
SNPs: Single nucleotide polymorphisms
SSr: Simple sequence repeat
