# Supplementary figures and images for: Transcriptome profiling and validation of gene based single nucleotide polymorphisms (SNPs) in sorghum genotypes with contrasting responses to cold stress
Source: BMC Genomics. 2015 Dec 9;16:1040. doi: 10.1186/s12864-015-2268-8 (PMC4673766; doi:10.1186/s12864-015-2268-8)

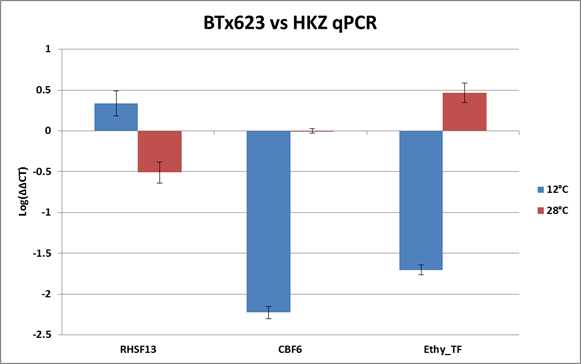

Supplement: Additional file 2: — Quantitative PCR plots of the selected expressed genes for technical validation. (TIF 114 kb) [file 12864_2015_2268_MOESM2_ESM.tif]

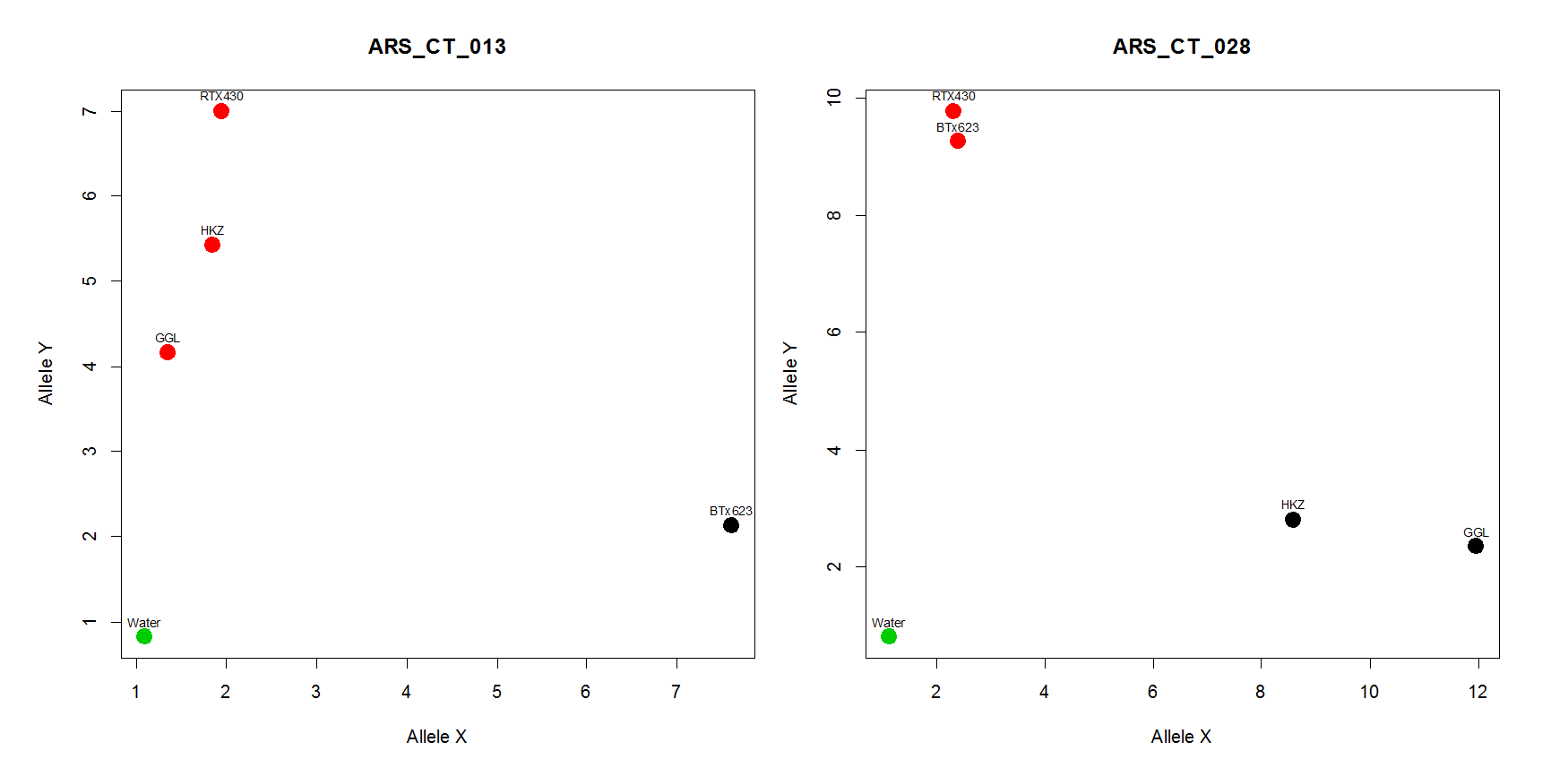

Supplement: Additional file 7: — An example of validation of SNPs between the sequenced accessions using KASP technology. (TIF 307 kb) [file 12864_2015_2268_MOESM7_ESM.tif]
