# Supplementary material for: Transcriptome profiling and validation of gene based single nucleotide polymorphisms (SNPs) in sorghum genotypes with contrasting responses to cold stress
Source: BMC Genomics. 2015 Dec 9;16:1040. doi: 10.1186/s12864-015-2268-8 (PMC4673766; doi:10.1186/s12864-015-2268-8)
Supplement: Additional file 6: — Primer sequences for validation of bi-allelic variant calls. (DOCX 15 kb) [file 12864_2015_2268_MOESM6_ESM.docx]

Additional File 6: Primer sequences for validation of bi-allelic variant calls.

| Primer Name | Common_Locus_Primer | Allele_Specific_Primer_1 | Allele_Specific_Primer_2 |
| --- | --- | --- | --- |
| ARS_Sb_CT_001 | ATGAAATCAGCCAGGAGCAC | AAGTCCTCGAAAACATTGCAG | AAGTCCTCGAAAACATTGCAT |
| ARS_Sb_CT_002 | TACAGCTACGCCTACGACGA | AGGTGACGGTGTAGTCTGTGG | AGGTGACGGTGTAGTCTGTGC |
| ARS_Sb_CT_003 | GAGGAGACGTTCGGGATCA | AGCTTGGGCTCAGCGAT | AGCTTGGGCTCAGCGAC |
| ARS_Sb_CT_004 | AGGGTTTAGGCTGTGGCAAT | AACCACTTCCAAATCCACAACT | CCACTTCCAAATCCACAACC |
| ARS_Sb_CT_005 | TGATTGCTAAGGGGGAAAAA | GCGTCAATTCCTCCTCCATA | GCGTCAATTCCTCCTCCATC |
| ARS_Sb_CT_006 | AGACGACGACGACGAAGG | GACGCCGTCCACCAA | TGACGCCGTCCACCAC |
| ARS_Sb_CT_007 | CGGAGAGGAATCAGAAGACCT | GCAGACGTCCGTGAAGG | TGCAGACGTCCGTGAAGA |
| ARS_Sb_CT_008 | CCAGAAACTGTTTGCACCAA | GCTGGCTTCAACTTCTGGG | GCTGGCTTCAACTTCTGGA |
| ARS_Sb_CT_009 | TGTTTTTCCATCTCCGGCTA | CGAATGGGCTTGGAGTTG | CCGAATGGGCTTGGAGTTA |
| ARS_Sb_CT_010 | GGGGCAATTTTGAATGCTTA | GTTGAAAAGGTAGAAGAGAACAGGAG | TTGAAAAGGTAGAAGAGAACAGGAA |
| ARS_Sb_CT_011 | TGCTCTACCATCCCTGGATAA | CATAATGATACTTCAGGAACTCCAA | CCATAATGATACTTCAGGAACTCCAC |
| ARS_Sb_CT_012 | ACAAATCTGCCGGGAAACTA | CCCCATCGGTTCCCG | ATCCCCATCGGTTCCCT |
| ARS_Sb_CT_013 | TGTAATCTGCCCCTTTGTATCC | TGAGACAGAACCGATCTACAAAGA | ATGAGACAGAACCGATCTACAAAGT |
| ARS_Sb_CT_014 | TCATTTCCAGTTCCACCAAA | ATGAATGACAAAGTGGATGCTTTA | GAATGACAAAGTGGATGCTTTC |
| ARS_Sb_CT_015 | AGGTCAGGCATCCGGTCT | CGAGGCGTGGACTCTGG | CGAGGCGTGGACTCTGC |
| ARS_Sb_CT_016 | GGTCAGCGTCTCCTTCATGT | GATGGACAGGACCACTATTTCAG | GATGGACAGGACCACTATTTCAA |
| ARS_Sb_CT_017 | CGAAAGGCAGAACACTCACA | TCTCTCCGATTGCTGAAACATA | CTCTCCGATTGCTGAAACATT |
| ARS_Sb_CT_018 | GTGCGCCCTAATAGACTGCT | TTGGGGGAATGATGAAACAG | TGGGGGAATGATGAAACAA |
| ARS_Sb_CT_019 | CGGAGGGAACGTAGTAGCC | GCAGACTGCATCGTCCAA | GCAGACTGCATCGTCCAC |
| ARS_Sb_CT_020 | TACGAGGCCGTCGTGTACTA | TTTTCCCAGACCTGTCGGT | TTCCCAGACCTGTCGGC |
| ARS_Sb_CT_021 | GTTCAGGTTGCGGTATTGGT | ACTGGCTACAAGTGTTCCTGG | CACTGGCTACAAGTGTTCCTGA |
| ARS_Sb_CT_022 | AGCCCTGCAGTTACAAAACC | TCACGGAAGAGCAGGACG | TTCACGGAAGAGCAGGACA |
| ARS_Sb_CT_023 | CATAGGCGTCGTCAGTGTTG | CATCGACCTCTCACCAAACA | ACATCGACCTCTCACCAAACT |
| ARS_Sb_CT_024 | ACGCTCTGCTTCCGCTTG | CGGCGCATAAGACGG | CCGGCGCATAAGACGA |
| ARS_Sb_CT_025 | TCTTTGATTGGGTTTCCAGTG | CGAGTCTAAGAATCACGGAAAGTG | CGAGTCTAAGAATCACGGAAAGTT |
| ARS_Sb_CT_026 | TGGCAGTCAGTTAGGAAGCA | CGTGATCTTCTGACCTGGACT | CGTGATCTTCTGACCTGGACC |
| ARS_Sb_CT_027 | ACGTCATGCTTCCTGACCTC | CATGCTCTTGGACGATGG | CATGCTCTTGGACGATGC |
| ARS_Sb_CT_028 | GGAAGGAAAGAAAAACTGGAGA | AAACTTTCCTCTTTGTGCTCTTG | CAAACTTTCCTCTTTGTGCTCTTC |
| ARS_Sb_CT_029 | AGGCCAGGTAGCAACAGATG | GAGGATCATCAGCCCCAT | GAGGATCATCAGCCCCAC |
| ARS_Sb_CT_030 | TAGTCAGGCGACTCCACCTC | CGCGCCAGTGGAAGG | TCGCGCCAGTGGAAGT |
