# Supplementary material for: Transcriptome profiling and validation of gene based single nucleotide polymorphisms (SNPs) in sorghum genotypes with contrasting responses to cold stress
Source: BMC Genomics. 2015 Dec 9;16:1040. doi: 10.1186/s12864-015-2268-8 (PMC4673766; doi:10.1186/s12864-015-2268-8)
Supplement: Additional file 8: — Description of the SNP position and SNP features on the sorghum chromosomes and validation of the bioinformatics allele calls on the sequenced lines and in two additional sorghum Germplasm RTx430 and GGL (PI567946). (DOCX 17 kb) [file 12864_2015_2268_MOESM8_ESM.docx]

Additional File 8: Description of the SNP position and SNP features on the sorghum chromosomes and validation of the bioinformatics allele calls on the sequenced lines and in two additional sorghum Germplasm RTx430 and GGL (PI567946).

| Primer | Chromosome | Position | Ref_Allele | Var_Allele | Ref_AA | Var_AA | BTx623 | GGL | RTx430 | HKZ |
| --- | --- | --- | --- | --- | --- | --- | --- | --- | --- | --- |
| ARS_Sb_CT_001 | chromosome_1 | 5567222 | T | G | S | T | 0 | 0 | 1 | 1 |
| ARS_Sb_CT_002 | chromosome_1 | 43997452 | C | G | I | V | 0 | 0 | 1 | 1 |
| ARS_Sb_CT_003 | chromosome_1 | 58160580 | T | C | L | P | 0 | 0 | 1 | 1 |
| ARS_Sb_CT_004 | chromosome_2 | 1286382 | T | C | K | N | 0 | 1 | 1 | 1 |
| ARS_Sb_CT_005 | chromosome_2 | 14393547 | C | A | L | V | - | - | - | - |
| ARS_Sb_CT_006 | chromosome_2 | 60660555 | A | C | A | V | 0 | 1 | 0 | 1 |
| ARS_Sb_CT_007 | chromosome_3 | 3499460 | G | A | I | V | 0 | 1 | 0 | 1 |
| ARS_Sb_CT_008 | chromosome_3 | 9735505 | A | G | V | I | 0 | 1 | 0 | 1 |
| ARS_Sb_CT_009 | chromosome_3 | 60616146 | G | A | K | R | 0 | 1 | 1 | 1 |
| ARS_Sb_CT_010 | chromosome_4 | 12244916 | A | G | P | T | 0 | 0 | 0 | 1 |
| ARS_Sb_CT_011 | chromosome_4 | 55960040 | C | A | P | Q | 0 | 0 | 0 | 1 |
| ARS_Sb_CT_012 | chromosome_4 | 66391485 | G | T | D | V | 0 | 1 | 0 | 1 |
| ARS_Sb_CT_013 | chromosome_5 | 418147 | A | T | F | L | 0 | 1 | 1 | 1 |
| ARS_Sb_CT_014 | chromosome_5 | 45153076 | C | A | G | A | 0 | 1 | 0 | 1 |
| ARS_Sb_CT_015 | chromosome_5 | 61284523 | G | C | R | K | 0 | 1 | 1 | 1 |
| ARS_Sb_CT_016 | chromosome_6 | 814724 | G | A | D | E | 0 | 1 | 1 | 1 |
| ARS_Sb_CT_017 | chromosome_6 | 2666890 | A | T | V | I | 0 | 1 | 1 | 1 |
| ARS_Sb_CT_018 | chromosome_6 | 47511826 | G | A | Q | H | 0 | 1 | 0 | 1 |
| ARS_Sb_CT_019 | chromosome_7 | 324684 | A | C | T | A | 0 | 1 | 0 | 1 |
| ARS_Sb_CT_020 | chromosome_7 | 8143329 | T | C | D | G | 0 | 1 | 0 | 1 |
| ARS_Sb_CT_021 | chromosome_7 | 58759745 | A | G | E | K | 0 | 1 | 1 | 1 |
| ARS_Sb_CT_022 | chromosome_8 | 2432668 | G | A | N | Y | - | - | - | - |
| ARS_Sb_CT_023 | chromosome_8 | 13353954 | A | T | A | T | 0 | 1 | 0 | 1 |
| ARS_Sb_CT_024 | chromosome_8 | 52451386 | G | A | E | A | - | - | - | - |
| ARS_Sb_CT_025 | chromosome_9 | 869075 | T | G | S | G | 0 | 1 | 0 | 1 |
| ARS_Sb_CT_026 | chromosome_9 | 49014069 | T | C | A | P | 0 | 0 | 0 | 1 |
| ARS_Sb_CT_027 | chromosome_9 | 58659331 | C | G | R | T | 0 | 1 | 0 | 1 |
| ARS_Sb_CT_028 | chromosome_10 | 5960968 | C | G | M | T | 0 | 1 | 0 | 1 |
| ARS_Sb_CT_029 | chromosome_10 | 17986326 | T | C | S | A | 0 | 0 | 1 | 1 |
| ARS_Sb_CT_030 | chromosome_10 | 51517061 | T | G | - | - | 0 | 1 | - | 1 |
